# Supplementary material for: Comparison of Methods for Incorporating Related Data When Developing Clinical Prediction Models: A Simulation Study
Source: Biom J. 2026 Aug 27;68(5):e70166. doi: 10.1002/bimj.70166 (PMC13520826; doi:10.1002/bimj.70166)
Supplement: Supplementary file 2 [file BIMJ-68-e70166-s001.zip › third submission code review - R2/RR-checklist-for-authors-2024-Haya Elayan.pdf]

Thank you for submitting your work for publication in Biometrical Journal. Before you resubmit your revised manuscript and supplement, we would like to ask you to carefully read through the following checklist to make sure that your mandatory code and data supplement complies with our standards for computational reproducibility.

If you prefer concrete examples over a checklist, please refer to Section 5 for links to published articles which follow best practices in dealing with frequently encountered challenges.
If you have any questions on how top prepare your supplement, please contact one of the RR editors Roman Hornung (`hornung@ibe.med.uni-muenchen.de`), Michael Kammer (`michael.kammer@meduniwien.ac.at`) or Lien Le (`le.dunglien@gmail.com`).

1 MAIN POINTS

- [x] We have verified that re-running the supplement's code on the supplement's data according to the instructions in the included `README` file (see Section 3) reproduces *all* figures, tables and results in the submitted article and its supplementary material.
  Please refer to Section 2 for details.
  Please actually do this before submitting – let one of your co-authors try to reproduce the results on their own machine with your supplement.
- [x] We have revised, cleaned up and documented the code files in this supplement to make sure that they follow commonly accepted standards for scientific computing.
  Please refer to Section 4 for details.
- [x] The code and data supplement has been uploaded to ManuscriptCentral as a single zip file containing all the scripts, programs, data files, intermediate results and a `README` file. Large data or results files that surpass ManuscriptCentral's file size limits are available from external repositories and linked to and documented in the `README` file.

2 REPRODUCIBILITY

- [x] The code and data supplement contains *all* code and data needed to reproduce *all* figures, tables and other results in the article and its supplementary material.
- The code files make it **as easy as possible to reproduce the results** in the manuscript:
  - [x] For simulation scripts that need to be run repeatedly with multiple settings, the supplement includes master scripts or Makefiles that perform these iterations and collates and

saves the intermediate results of the separate runs with descriptive filenames in a separate results folder. Such "master scripts" can also be literate programming documents, i.e., Jupyter Notebooks, Sweave, R Markdown, etc.

- [x] Reproducing our simulation results does not require repeated manual edits to the simulation code itself.
- [x] The supplement does not include multiple almost-identical copies of the same simulation scripts with slight modifications for the different simulation settings or different methods being compared (no "copy-paste"-programming). See Section 4.

- The code files make it **as easy as possible to associate the outputs** of the code with the results in the manuscript:
  - [x] They include comments clearly stating which figure or table in the manuscript is produced by which section of code.
  - [x] They create dataframes or tables with identical structure, column names, and row names as the tables in the manuscript and automatically save them with unique, descriptive filenames in a separate results folder.
    Filenames that correspond to the numbering of the tables in the manuscript and supplementary material are preferred.
  - [x] They save graphical output automatically in a separate results folder with unique, descriptive filenames.
    Filenames that correspond to the numbering of the figures in the manuscript and supplementary material are preferred.

- Randomization results are reproducible:
  - [x] Any code whose output relies on results of a random number generator (RNG) is initialized by setting the seed for the RNG so that results are exactly reproducible and are identical to the results contained in the manuscript and its supplementary material.
  - [x] By re-running any code that relies on results of a random number generator (RNG) multiple times, we have verified that the Monte Carlo errors of the summarized results are negligible and do not affect any of the substantive conclusions drawn from them (e.g., rank orders of different methods according to the relevant performance criteria do not change when simulation studies are re-run).

- If data sets are not allowed to be published or shared: (*check all that apply*)
  - [x] Synthetic or suitably anonymized pseudo-data comparable to the original data in size and structure have been included.

- ☐ The original data has been made available to RR editors strictly for the purpose of the reproducibility audit in a separate file.

- If the computations required for reproduction of the article's results run for more than a couple of hours on a standard desktop PC:

  - ☑ The code supplement provides intermediate results and the scripts used to produce the figures/tables from them and information on which parameters to change to reduce the run time, where applicable. If files containing intermediate results are too large to upload to ManuscriptCentral or e-mail, please upload them to a data repository (Zenodo, Figshare, etc) or share them via a file hoster[1]. If results files cannot be made publicly available, we can set up a secure file sharing link as well. Please contact the RR editors if required.
  - ☑ We have verified that the code and intermediate results in the supplement enable spot checks of reproducibility for specific settings/replications without the need to re-run the entire simulation study. The code and its saved intermediate results are structured so that it is easy to quickly re-run specific parts of the experiment and verify that the results are identical to the saved intermediate results supplied by the authors.

3 DOCUMENTATION

The `README` file …

- ☑ is a `.txt`, HTML or PDF file.
- ☑ contains version information for all the software (i.e., operating system version and version info for statistical libraries, utilities, compilers, and add-on packages, whatever is applicable) used anywhere in the scripts in the supplement. For R: contains the output of `sessionInfo()` after loading all packages that are required anywhere in the supplement.
  Alternatively, authors can provide a reproducible computation environment in the form of a Docker container, a `renv`, `checkpoint` or `packrat` snapshot for `R`, etc. For R packages only available on Github etc, please also provide the relevant commit hash (or release tag, preferably) and installation instructions.
- ☑ explains which scripts to run in which order in order to reproduce which of the figures and tables in the paper and its supplements.
  (This is not necessary if the supplement contains a suitably documented master script or uses a dependency graph system like `R`'s `drake` or `targets` packages or a `Makefile`.)

[1] … one that does not require a user account for access, i.e., *not* OneDrive

- [x] clearly describes any manual alterations to the code required for reproducing the results, with reference to the respective file names, line numbers, and the exact content of the necessary edits. **If at all possible, such manual edits must be avoided** by including suitable master scripts or Makefiles instead.
- [x] contains full documentation for all data sets including their provenance, information about intellectual property holders or licensing terms, and (links to) data dictionaries defining their content.
- [x] contains a listing of the files and folder structure with brief explanations of their content.

4 CODING STANDARDS

- [x] All source code is submitted in ASCII files, preferably in UTF-8 encoding.
- [x] All names of files, functions, macros and variables and all code comments are in English.
- [x] All code files use a consistent, sensible format and style with
  - proper spacing (spaces after commas and around operators, etc.),
  - human-readable line lengths (max. 80-100 characters per line),
  - semantically correct & consistent indentation.
- [x] Code files are organized well:
  - Code is split into separate files according to functionality with informative, descriptive names.
  - The supplement uses a sensible folder structure with informative, descriptive names.
  - R script files are saved in files with file extension `.r` or `.R`, SAS scripts as `.sas`, Stata programs as `.ado` or `.do`, etc. Code is never saved in `.txt` files, PDFs, word processor documents and similar file formats.
- [x] All functions and macros defined in the code supplement are properly documented with text definitions of all their inputs and outputs.
- [x] The supplement does not include multiple almost-identical copies of scripts with slight modifications to iterate through different settings. Instead, code reused multiple times is contained in suitably defined functions or macros, which are repeatedly called for the different settings. **The supplement does not contain error-prone, excessively long and unreadable "copy-paste" code.** This also means using for-loops or other iterators (in R: `Map()`, `apply()`, etc) to iterate over settings instead of copying the same command with slightly different arguments for each setting many times.

- [x] For simulation experiments that need to be run repeatedly with different settings, the supplement either includes master scripts or Makefiles that perform these iterations and collate and save the intermediate results of the separate runs with informative, descriptive file names, or the simulation code itself performs replication, collection, and saving of results in a transparent, well documented manner. In either case, **reproducing the simulation results does not require repeated manual edits to the simulation scripts themselves, nor any manual collection of results from console output.**

- [x] Code files do not contain a mixture of code that performs the analysis with code that defines the functions or macros used in the analysis. Code files are split into a) files providing function or macro definitions and b) scripts that load these definitions (in R: via `source()`) and run the analyses or simulations.

- [x] Code files do not contain extraneous or outdated code. All scripts and function definitions only contain code relevant for reproducing the published results. No lines that are commented out nor code that is not actually used is contained in the supplement.

- [x] Code files do not contain commands that affect users' global workspace or software installation unnecessarily. They don't automatically (re-)install packages, only load them. They do not delete the entire user workspace (in R: no `rm(list= ls())`) and do not set or modify any system variables.

- [x] Code files do not contain any absolute paths and only use paths relative to the current file or working directory, if the latter is specified in your `README` and script.
  (i.e., no paths like `C:/Dropbox/BiomJ-paper/Final Revision/My Code.R` , use paths like `./simulation_1.R` or `../config/params.txt` instead). If this is impossible, the `README` file provides file names and line numbers of specifically where and how to change any absolute paths .

- [x] Code is as platform-independent as possible. In R, avoid OS-specific commands such as `windows()` or `windowsFonts()` and use `file.path()` to make folder separators platform-independent. If the code supplement contains R packages, they should not be submitted as binaries (i.e., `.zip` -files) but as package bundles (file ending `.tar.gz`) or source packages instead . Mac and Windows users should verify that capitalization in all file paths is correct so that they work as intended for UNIX-based operating systems which have case-sensitive paths.

- For code in compiled languages (C, C++, FORTRAN, etc.):

  - [ ] The supplement includes both the documented source code and suitable `Makefile` s or complete compilation and installation instructions, as well as pre-compiled executables.

5 EXEMPLARY SUPPLEMENTS

Some links to accepted manuscripts which largely follow best practices and deal with frequently encountered challenges:

- a well structured supplement for a manuscript containing both real data analyses and fairly complex simulations, which also provides intermediate results for computationally intensive simulation experiments:
  Günther, F., Brandl, C., Heid, I. M., & Küchenhoff, H. (2019). Response misclassification in studies on bilateral diseases. *Biometrical Journal*, **61**(4), 1033–1048. Supplement (30 MB)
- a supplement using `Rmarkdown` files to simplify reproduction of results, with optional re-loading of author-supplied intermediate results for computationally intensive steps:
  Kopp-Schneider, A., Calderazzo, S., & Wiesenfarth, M. (2020). Power gains by using external information in clinical trials are typically not possible when requiring strict type I error control. *Biometrical Journal*, **62**(2), 361–374. Supplement (1 MB)
- a well structured supplement for a manuscript for which only synthetic pseudo-data is publicly available due to data sharing restrictions:
  Heller, G.Z., Couturier, D.-L., Heritier, S.R (2019). Beyond mean modelling: Bias due to misspecification of dispersion in Poisson-inverse Gaussian regression. *Biometrical Journal*, **61**(2), 333–342 Supplement (7 MB)
- a well structured supplement providing large data sets from an external source:
  Rui, R., & Tian, M. (2021). Joint estimation of case fatality rate of COVID-19 and power of quarantine strategy performed in Wuhan, China. *Biometrical Journal*, **63**(1), 46–58. Supplement (2.4 MB)
